# Human Ophthalmomyiasis Interna Caused by *Hypoderma tarandi,* Northern Canada

**DOI:** 10.3201/eid1401.070163

**Published:** 2008-01

**Authors:** Philippe R.S. Lagacé-Wiens, Ravi Dookeran, Stuart Skinner, Richard Leicht, Douglas D. Colwell, Terry D. Galloway

**Affiliations:** * University of Manitoba, Winnipeg, Manitoba, Canada; † Agriculture and Agri-food Canada, Lethbridge, Alberta, Canada

**Keywords:** Myiasis, ophthalmology, arctic regions, ivermectin, treatment, review, Canada, dispatch

## Abstract

Human myiasis caused by bot flies of nonhuman animals is rare but may be increasing. The treatment of choice is laser photocoagulation or vitrectomy with larva removal and intraocular steroids. Ophthalmomyiasis caused by *Hypoderma* spp. should be recognized as a potentially reversible cause of vision loss.

Ophthalmomyiasis interna is invasion of the globe by larvae of any species of oestrid flies; ophthalmomyiasis externa involves only the external ocular structures (*1*). *Dermatobia hominis,* endemic to tropical or subtropical areas, and *Oestrus ovis* (sheep bot fly) cause most cases of ophthalmomyiasis (*2*). Both typically cause ophthalmomyiasis externa (*2*). Only 2 bot flies inhabit Nearctic circumpolar regions: the Caribou bot fly (*Hypoderma tarandi*) and the Caribou nasal bot fly (*Cephenemyia trompe*), a nonhuman pathogen (*2*). *H. tarandi* is a nonbiting fly whose obligate endoparasitic larvae typically affect caribou throughout their circumpolar range (*2**,**3*). From late June to early September. the fly lays eggs directly on the guard hairs of the caribou (*2**,**3*). Once deposited, eggs hatch into larvae that penetrate skin (*3*). They move subcutaneously to reach the animal’s dorsal region, where they cut breathing holes and are encased within granulomatous cysts, termed warbles. There they develop for 9–11 months; in May or June of the following year, they leave the animal and pupate on the ground (*3*). Mated adult females are capable of long flights (≈900 km) in search of suitable hosts (*3*).

Infestations by *H. tarandi* in humans are rare but are likely underreported (*4*). Related species, *H. bovis* and *H. lineatum,* inhabit various regions of North America, Europe, and Asia and have also been implicated in human disease (*2**,**5*). A third species that affects cattle and yaks in China (*H. sinense*) is also responsible for human infestations (*2*). The pathophysiology of human ophthalmomyiasis by *Hypoderma* spp. is not known. Eyebrows and eyelashes have been suggested as possible targets for oviposition (*3*). Oviposition on human scalp hair has been achieved experimentally and could be the preferential site in humans (*3*). An alternative explanation is transfer of the larvae directly from the guard hairs of the caribou to the human eye or skin through close contact with animal pelts. The parasite does not appear to complete its life cycle in humans (*1**,**2*).

We present the first, to our knowledge, 2 published cases of ophthalmomyiasis interna caused by *H. tarandi* in Canada. Furthermore, we present the first published use of *Hypoderma* spp. serologic testing to assist in the diagnosis of myiasis in humans.

## The Cases and Literature Review

**Figure 1:**
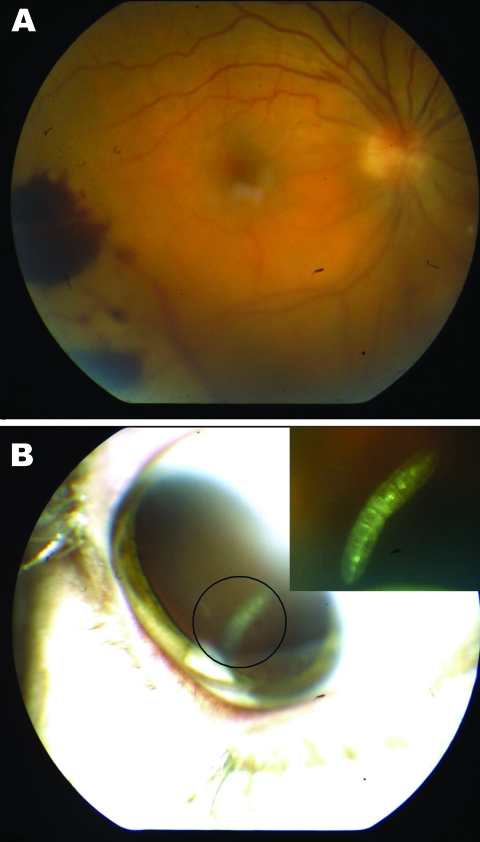
A) Retinal hemorrhages visible on funduscopic examination of right eye of a 41-year-old woman, Nunavut, Canada, with ophthalmomyiasis intern). B) Segmented 3-mm larva with a cylindrical body, no visible spines, and indistinguishable anterior and posterior ends in the vitreous cavity, corresponding to the first instar of *Hypoderma tarandi*.

The first patient was a 41-year-old woman from Rankin Inlet, Nunavut, Canada, who noticed floaters (objects in the field of vision that originate in the vitreous) in her right eye in August 2006. Initial funduscopic examination showed posterior vitreous detachment. Two weeks later, her vision was more impaired; repeat funduscopy showed panuveitis. Pretreatment blood count was within normal limits with no eosinophilia. Topical steroids were ineffective. At a third assessment, her visual acuity was 20/400; funduscopic examination showed an intraocular larva (Figure 1). The parasite appeared to recede behind the retina and could not be photocoagulated. A pars plana vitrectomy and intraocular laser treatment of the entry and exit sites were performed. Triamcinolone (0.4 mg) was administered for intraocular inflammation, and antibiotics were given as prophylaxis. The larva was not recovered. Postoperative magnetic resonance imaging demonstrated no parasite or abnormality. The larva was assumed to be that of *H. tarandi* because of its appearance (shape, size, and segments), the late summer timing, and the patient’s residence in the subArctic. Serologic testing for *H. tarandi* by Western blot, as described by Baron and Colwell, was performed (*6*). Six weeks after symptom onset, serum was positive for immunoglobulin (Ig) G, IgE, and IgM to hypodermin C, a larval collagenase of *H. tarandi* (*7*)*.* No seroreactivity to the hypodermins A and B of *H. lineatum* was observed. Because the larva was unrecoverable, the patient was treated with 1 oral dose (9 mg) of ivermectin (Merck & Co., Kirkland, Quebec, Canada) 1 week after vitrectomy. After 6 months, her visual acuity had improved to 20/30.

**Figure 2:**
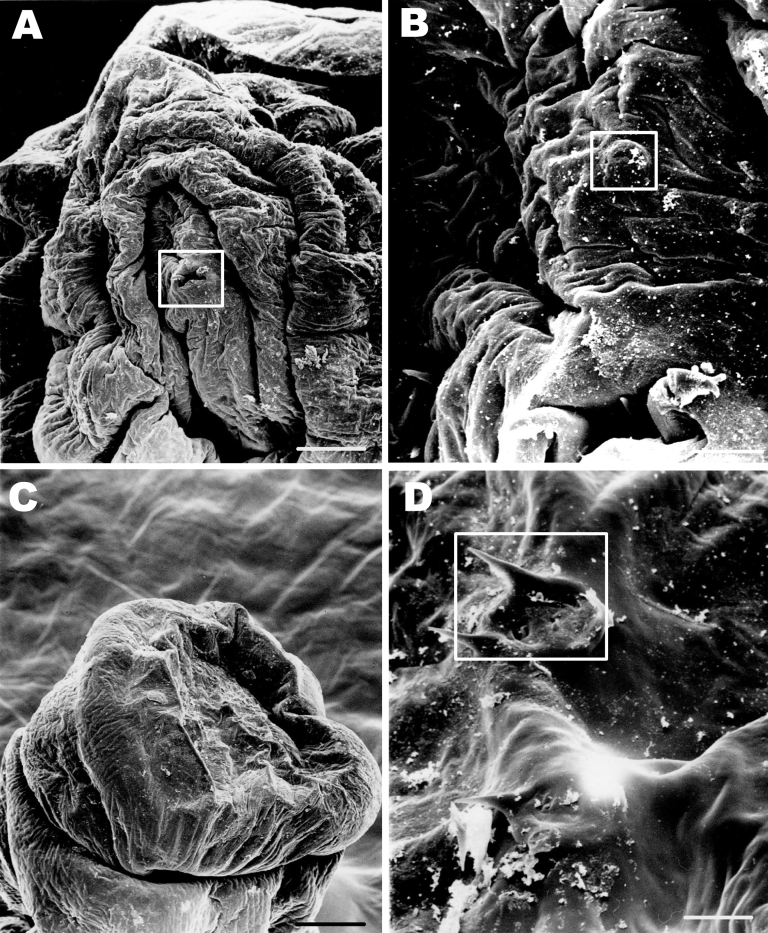
Scanning electron microscope images of the parasite from an 11-year-old Inuit boy, Nunavut, Canada. A) Anterior end of the maggot. The cephalic segment is evident; mouth and mouth hooks are present (boxed). Scale bar = 50 μm. B) The characteristic cephalic sensory array (boxed). Scale bar = 10 μm. C) Posterior segments of the maggot. Scale bar = 100 μm. D) Spiracular openings on the posterior segments of the maggot characteristic of first instar of *Hypoderma*. Scale bar = 10 μm.

The second patient was an 11-year-old Inuit boy from Chesterfield Inlet, Nunavut, Canada, who was examined in October 1997 because of a painful right eye, scleral redness, and blurry vision. Examination found uveitis and glaucoma. Topical steroid therapy was begun, after which retinal detachment, hemorrhages, and a 4-mm larva in the subretinal space were noted. Blood work showed marginal eosinophilia; computed tomographic examination of the brain and orbits showed no abnormalities. The larva was removed by pars plana vitrectomy and retinotomy. Examination of the larva by dissection microscopy showed a 4 × 1–mm larva. Scanning electron microscopy, performed according to Colwell et al. (*8*), confirmed the characteristic of the first instar of *Hypoderma* spp., i.e., mouth hooks, anterior sensory structure, spines, and spiracles (Figure 2). The presence of only 2 lateral spines per spiracle indicated *H. tarandi*. Western blot testing showed the presence of IgG only to hypodermin C. When asked about his exposure to caribou, the boy admitted to tracking, hunting, and skinning caribou. The patient’s disease progressed despite therapy, and his eye was enucleated. Pathologic examination showed retinal detachment, eosinophilic choroiditis, and retinitis. Chronic nongranulomatous inflammation was noted in the ciliary body and iris. No additional larvae were found.

We searched the literature, using PubMed, for the terms “ophthalmomyiasis” with limits “human” and “English.” We also reviewed references of selected publications. We reviewed only cases of ophthalmomyiasis interna caused by oestrid flies found in North America, which were confirmed by visible larvae (Appendix Table). Demographics and clinical presentation of patients are in the Table. For statistical analysis, outcomes were separated into good (vision unchanged from baseline or better than 20/80), moderate (able to see shapes or fingers or vision worse than 20/80 but not blind), and severe (able only to see light or movement, completely blind or enucleated). Although moderate or severe vision loss appeared to occur more commonly with *Hypoderma* than *Cuterebra* infestations (53.3% vs 14.3%), this frequency was not statistically significant (p = 0.08). Photocoagulation appeared to produce better outcomes (80% good outcomes) than no intervention (73.7% good outcomes) or surgical removal/vitrectomy (41.2% good outcomes); however, χ^2^ analysis showed no statistically significant difference (p = 0.121). This observation may suggest that by the time the patient seeks treatment, damage to ocular structures has already occurred. Recent cases have been treated with photocoagulation or vitrectomy and intraocular steroid administration. Although no specific intervention is associated with better outcomes, given the difficulty in studying outcomes in this rare condition and the success of this therapy for other foreign bodies in the eye, this course of action is reasonable. Ivermectin as medical therapy for ophthalmomyiasis interna has not been reported, but it is effective as therapy and prophylaxis for bot fly infestation of livestock (*9*). Ivermectin as adjunctive therapy in select cases of ophthalmomyiasis may be of benefit, but evidence is lacking. Only 1 case of ophthalmomyiasis externa caused by *D. hominis* and treated with ivermectin has been reported (*10*).

**Table:** Demographics and clinical presentation of 32 patients with ophthalmomyiasis interna*

Characteristic	No. (%)
Symptom	
Red eye	21 (60)
Vision loss	24 (68)
Floaters	24 (68)
Eye pain	6 (17)
Scotomas	3 (9)
Sex	
Male	27 (77)
Female	7 (20)
Loss of vision	
Severe or total†	12 (34)
Moderate	2 (6)
Mild or none	19 (54)

*Patients identified during literature search and described in the Appendix Table. Mean patient age was 25.2 y; median, 16 y. †Includes blindness caused by enucleation.

## Conclusions

Human myiasis caused by bot flies of nonhuman animals is rarely reported. However, diagnoses may increase as a result of increasing population in northern latitudes, encroachment into habitats with natural hosts like caribou, and better access to ophthalmologists. The treatment of choice seems to be laser photocoagulation or vitrectomy with larva removal and coadministration of intraocular steroids. In areas where *Hypoderma* spp. are common, healthcare providers should consider this condition and promptly refer patients to an ophthalmologist.

## Supplementary Material

Appendix Table Reported cases of ophthalmomyiasis caused by North American oestrid flies (Diptera:Oestridae)
