# Supplementary material for: Human Ophthalmomyiasis Interna Caused by Hypoderma tarandi, Northern Canada
Source: Emerg Infect Dis. 2008 Jan;14(1):64–6. doi: 10.3201/eid1401.070163 (PMC2600172; doi:10.3201/eid1401.070163)
Supplement: Appendix Table — Reported cases of ophthalmomyiasis caused by North American oestrid flies (Diptera:Oestridae) [file 07-0163_appT-s1.pdf]

Appendix Table. Reported cases of ophthalmomyiasis caused by North American oestrid flies (Diptera:Oestridae)

Reference	Year of case	Patient age (y), sex	Fly species	Residence	Signs and symptons	Treatment	Vision loss
This Article	2006	41, F	*Hypoderma tarandi*	Canada	Visual loss, red eye.	Vitrectomy, steroids, ivermectin	Mild
(**1.**)	2003	3, F	*H. bovis*	Turkey	Painful red eye	Vitrectomy, larva removal	Lost to follow-up
(**2.**)	2000	41, M	*Cuterebra* spp.	USA	Blurred vision, scotoma	Photocoagulation, steroids	Mild
This article	1997	11, M	*H. tarandi*	Canada	Visual loss, red eye	Vitrectomy, removal of larva, steroids	Enucleation
(**3.**)	1997	53, M	Unknown	USA	Vision loss	Vitrectomy, larva removal	Severe
(**4.**)	1995	14, M	*Cuterebra* sp.	USA	Visual loss	Vitrectomy, removal of larva	Mild
(**5.**)	1995	16, M	Unknown	USA	Pain, red eye, vision loss	Photocoagulation of larva	Mild
(**6.**)	1990	50, M	*Cuterebra* sp.	USA	Scotoma, vision loss	Laser photocoagulation	Mild
(**7.**)	1988	31, M	Unknown	USA	None	Vitrectomy, removal of larva	Complete recovery
(**8.**)	1986	5, F	*H. tarandi*	The Netherlands	Pain, red eye, vision loss	Vitrectomy, silicone oil	Severe
(**9.**)	1986	77, M	*H. tarandi*	Sweden	Vision loss	Vitrectomy, removal of larva	Severe
(**10.**)	1985	10, M	*H. tarandi*	Norway	Red eye	Vitrectomy, steroids, larva removal	Moderate
(**10.**)	1985	43, M	*H. tarandi*	Sweden	Red eye, vision loss	Steroids, vitrectomy, larva removal	Complete recovery
(**11.**)	1984	2, F	*Hypoderma* spp.	USA	Red eye	Vitrectomy	Mild
(**12.**)	1984	16, F	Unknown	USA	Vision loss, pain, red eye	Lidocaine, laser coagulation	Complete recovery
(**10.**)	1983	9, M	*H. tarandi*	Norway	Red eye, vision loss	Vitrectomy, steroids	Enucleation
(**13.**)	1983	33, M	*Cuterebra* sp.	USA	Red eye	Steroids	Complete recovery
(**14.**)	1983	9, M	*H. tarandi*	Norway	Visual loss, red eye	Vitrectomy	Enucleation
(**10.**)	1981	7, M	*H. tarandi*	Norway	Red eye, vision loss	Vitrectomy, larva removal, steroids	Nearly complete blindness
(**15.**)	1981	33, M	Unknown	USA	Vision loss	Steroids	Nearly complete blindness
(**16.**)	1981	10, M	Suspected *H. lineatum*	USA	Vision loss, scotomas	Vitrectomy, steroids	Unilateral blindness

(**17.**)	1981	11, M	*Cuterebra* sp.	USA	Eye pain, reduced vision	Vitrectomy, larva removal	Nearly complete blindness
(**15.**)	1980	13, M	*H. tarandi*	Norway	Vision loss, floaters	Vitrectomy, removal of larva	Mild vision loss
(**15.**)	1980	15, M	Unknown	USA	Vision loss	None	Mild vision loss
(**15.**)	1979	3 (sex unknown)	Unknown	USA	Asymptomatic	None	Complete recovery
(**15.**)	1979	49, M	Unknown	USA	Floater, vision loss	None	Major vision loss
(**15.**)	1974	21, M	Unknown	USA	Painful red eye	Steroids, thiabendazole, laser photocoagulation	Moderate vision loss
(**15.**)	1969	43, F	*Cuterebra* sp.	USA	Floater	None	Complete recovery
(**15.**)	1967	45, F	*Cuterebra* sp.	USA	Pain, red eye, vision loss.	Steroids, atropine	Mild vision loss
(**16.**)	1967	7, M	*H. lineatum*	USA	Red eye, vision loss	Vitrectomy, removal of larva, steroids	Unilateral blindness
(**17.**)	1945	12, M	Presumed *H. tarandi*	Norway	Red eye	None	Complete recovery
(**10.**)	1943	50, M	Presumed *H. tarandi*	Sweden	Painful red eye	None	Complete recovery
(**18.**)	1938	6, M	*Hypoderma* sp.	USA	Painful red eye	Pilocarpine, vitrectomy, larva removal	Not reported
(**19.**)	1934	46, M	Unknown	USA	Pain, red eye, vision loss	None	Complete recovery
(**20.**)	1933	48, M	Unknown	USA	Blindness, red eye	Atropine	Blindness

References

1. Gozum N, Kir N, Ovali T. Internal ophthalmomyiasis presenting as endophthalmitis associated with an intraocular foreign body. Ophthalmic Surg Lasers Imaging. 2003;34:472–4.
2. Buettner H. Ophthalmomyiasis interna. Arch Ophthalmol. 2002;120:1598–9.
3. Jakobs EM, Adelberg DA, Lewis JM, Trpis M, Green WR. Ophthalmomyiasis interna posterior. Report of a case with optic atrophy. Retina. 1997;17:310–4.
4. Glasgow BJ, Maggiano JM. Cuterebra ophthalmomyiasis. Am J Ophthalmol. 1995;119:512–4.
5. Phelan MJ, Johnson MW. Acute posterior ophthalmomyiasis interna treated with photocoagulation. Am J Ophthalmol. 1995;119:106–7.
6. Currier RW, Johnson WA, Rowley WA, Laudenbach CW. Internal ophthalmomyiasis and treatment by laser photocoagulation: a case report. Am J Trop Med Hyg. 1995;52:311–3.
7. Laborde RP, Kaufman HE, Beyer WB. Intracorneal ophthalmomyiasis. Case report. Arch Ophthalmol. 1988;106:880–1.
8. Gryseels B, Mertens DA, Mehl R. An imported case of ophthalmomyiasis interna posterior in The Netherlands caused by a larva of the reindeer warble fly. J Infect Dis. 1991;163:931–2.
9. Gjotterberg M, Ingemansson SO. Intraocular infestation by the reindeer warble fly larva: an unusual indication for acute vitrectomy. Br J Ophthalmol. 1988;72:420–3.
10. Syrdalen P, Stenkula S. Ophthalmomyiasis interna posterior. Graefes Arch Clin Exp Ophthalmol. 1987;225:103–6.
11. Rapoza PA, Michels RG, Semeraro RJ, Green WR. Vitrectomy for excision of intraocular larva (Hypoderma species). Retina. 1986;6:99–104.

12. Forman AR, Cruess AF, Benson WE. Ophthalmomyiasis treated by argon-laser photocoagulation. Retina. 1984;4:163–5.
13. Newman PE, Beaver PC, Kozarsky PE, Waring GO III. Fly larva adherent to corneal endothelium. Am J Ophthalmol. 1986;102:211–6.
14. Kearney MS, Nilssen AC, Lyslo A, Syrdalen P, Dannevig L. Ophthalmomyiasis caused by the reindeer warble fly larva. J Clin Pathol. 1991;44:276–84.
15. Syrdalen P, Nitter T, Mehl R. Ophthalmomyiasis interna posterior: report of case caused by the reindeer warble fly larva and review of previous reported cases. Br J Ophthalmol. 1982;66:589–93.
16. Edwards KM, Meredith TA, Hagler WS, Healy GR. Ophthalmomyiasis interna causing visual loss. Am J Ophthalmol. 1984;97:605–10.
17. Custis PH, Pakalnis VA, Klintworth GK, Anderson WB Jr, Machemer R. Posterior internal ophthalmomyiasis. Identification of a surgically removed Cuterebra larva by scanning electron microscopy. Ophthalmology. 1983;90:1583–90.
18. O'Brien CS, Allen JH. Ophthalmyiasis interna anterior, report of *Hypoderma* larva in anterior chamber. Am J Ophthalmol. 1939;22:996–8.
19. Anderson W. Ophthalmomyiasis interna. Case report and review of the literature. Trans Am Acad Ophthalmol Otolaryngol. 1934;39:218–39.
20. DeBoe M. Dipterous larva passing from the optic nerve into the vitreous chamber. Arch Ophthalmol. 1933;10:824.
